# Supplementary material for: Assembly Formation of P65 Protein, Featured by an Intrinsically Disordered Region Involved in Gliding Machinery of Mycoplasma pneumoniae
Source: Biomolecules. 2025 Mar 17;15(3):429. doi: 10.3390/biom15030429 (PMC11940719; doi:10.3390/biom15030429)
Supplement: Supplementary file 1 [file biomolecules-15-00429-s001.zip › biomolecules-3502309-supplementary.pdf]

```
P65      1  MDINKPGWNQSDQQAT-AY----------DPNQQQYYGDGSTYYDPDQ------AVDPNQA
MG_217   1  MEKNRSAFQQNQQASNQPFNQDQNQYYQDPNQQQFNQSG---FDPNQQQFNQPGFDPNQQ

        45  YYPDPNTYPDAAAYYGYGQDGQAYPQDYAQDPNQAYYADPNAYQDPNAYTDPN--AYVDP
        58  YYQDPNQQQFNQA----------------GFDQNQQYYQDPNQQQFNQPGFDPNQQYYQDP

       103  NAYQDPNAYVDPNNY-TDPNAYYGYGQDGQAYPQDYAQDPNQAYYADPNAYQDPNAYTDP
       103  NQQQFNQAGFDQNQYYQDPNQQ-QFNQSGFDQNQ-YYQDPNQQQFNQPSFDLNNQ-----

       162  YYVTSTDPNAYYGQVDNVPALEASDLAYEVTPQEQAAEQELFSEPETKVIREIHEFPFEK
       156  --------------QFNQPGFNQS-PAFEITPQEQKAEQEMFGEEPPQVVREIHELPFEK

       222  IRSYFQTDFDSYNSRLTQLKDKLDNAIFSMRKAIDTVKENSANLQIMKQNFERQLKEQQT
       201  IRSFLQSDFDSYNFRLNSLKSKLDNALYSLDKTIQNTNENTANLEAIRHNLEQKIQN-QS

       282  QRLTSNTDAEKIGAKINQLEERMQRLSRTMESVEWTKKEPRQEQFDPRFVDPRNFNNYVN
       260  KQLRTNFDTQKLDDKINELEIRMQKLTRNFESLSELSKHNSYPNYYEKL--------LPN

       342  NTDTMMSMFEKVLMMNLLRSTTPVQPPVQYFTPQPLTASPRPVYEEPISASFRRRGYRGD
       312  GGDSMTNVFEKALMMNLLRTTLPPQPQVQYY-PQPYPY-IRPYYDEPIYAGFRRRGYR-D

       402  EFYE
       369  DFYE
```

Figure S1. Sequence similarity between P65 (upper) and an ortholog from *M. genitalium* (MG_217) (lower). Identical and similar amino acids are marked by black and gray boxes, respectively.

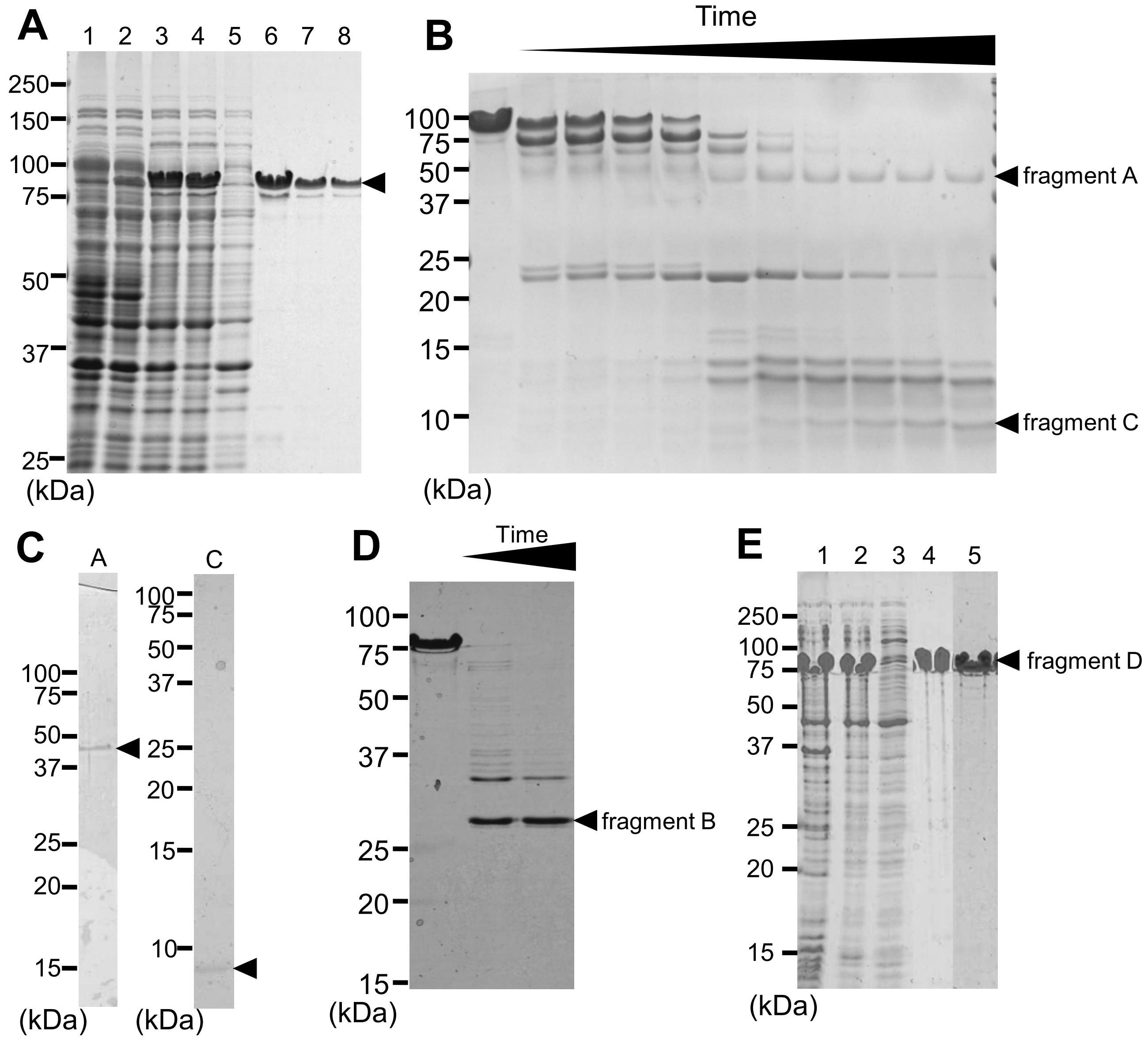

Figure S2. Isolation of P65 and fragments monitored through SDS-PAGE. The molecular masses are indicated on the left. (A) The full-length P65 was isolated with three steps as described in "Materials and Methods". Lane 1, *E. coli* lysate without plasmid; lane 2, *E. coli* lysate without IPTG; lane 3, *E. coli* lysate induced with IPTG to express P65; lane 4, supernatant of lysate with 6 M urea after centrifugation; lane 5, pellet of lysate with 6 M urea after centrifugation; lane 6, peak fraction of HisTrap HP column chromatography; lane 7, Refolded P65 by dialysis; and lane 8, peak fraction of gel filtration. Protein fractions were separated by SDS-PAGE with 10% polyacrylamide gels and gels were stained with Coomassie brilliant blue (CBB). The P65 band is marked by a black triangle. (B) Limited proteolysis of P65. Protein profiles of the isolated P65 digested by trypsin for various reaction times: 0, 1, 3, 5, 10, 30, 60, 90, 120, 150, and 180 min. The digested protein fractions were analyzed by SDS-PAGE with 10% polyacrylamide gels. The resultant peptide fragments were identified by PMF and fragments A and C were further analyzed. (C) Isolated fragments A and C. The focusing protein bands are marked by a triangle. (D) Limited proteolysis of P65. Isolated P65 was digested by endopeptidase Asp N for 0, 8, and 16 h and subjected to SDS-PAGE with 10% polyacrylamide gels. The resultant peptide fragment marked by a black triangle was identified as fragment B by PMF. The digest after 16 h was dialyzed and observed by EM. (E) Fragment D was isolated with three steps as described in Materials and Methods. Lane 1, *E. coli* lysate induced with IPTG to express fragment D; lane 2, soluble fraction after centrifugation; lane 3, insoluble fraction after centrifugation; lane 4 peak fraction of HisTrap HP column chromatography; and lane 5, peak fraction of gel filtration. Protein fractions were analyzed by SDS-PAGE with 10% polyacrylamide gels and stained with CBB. Fragment D is marked by a black triangle.

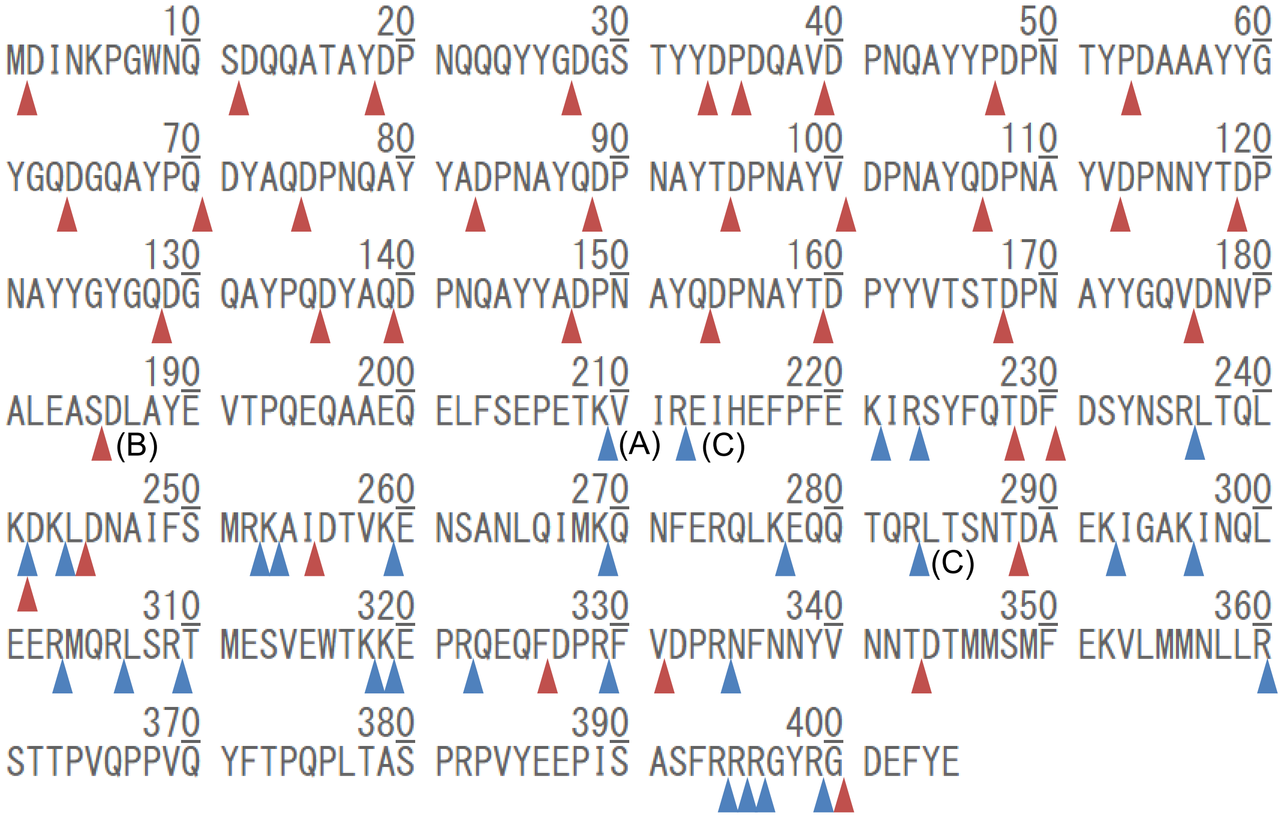

Fig S3. Protease cleavage sites in the amino acid sequence of P65. The sites for trypsin and endopeptidase Asp N are marked by blue and red triangles, respectively. The ends of fragments A, B, and C shown in Figure S2B are marked in the right of the triangles.

Fragment B

1 MDINKPGWNQ SDQQATAYDP NQQQYYGDGS TYYDPDQAVD PNQAYYPDPN
51 TYPDAAAYYG YGQDGQAYPQ DYAQDPNQAY YADPNAYQDP NAYTDPNAYV
101 DPNAYQDPNA YVDPNNYTDP NAYYGYGQDG QAYPQDYAQD PNQAYYADPN
151 AYQDPNAYTD PYYVTSTDPN AYYGQVDNVP ALEASDLAYE VTPQEQAAEQ
201 ELFSEPETKV IR**EIHEFPFE** **KIRSYFQTDF** **DSYNSRLTQL** **KDKLDNAIFS**
251 **MR**K**AIDTVKE** **NSANLQIMK**Q NFER**QLKEQQ** **TQR**LTSNTDA EKIGAKINQL
301 EERMQRLSR**T** **MESVEWTKKE** **PR**QEQFDPR**F** **VDPRNFNNYV** **NNTDTMMSMF**
351 **EKVLMMNLLR** STTPVQPPVQ YFTPQPLTAS PRPVYEEPIS ASFRR**RGYRG**
401 **DEFYE**

Unformatted sequence string: **405 residues** (for pasting into other applications).

Sort peptides by ◉ Residue Number ○ Increasing Mass ○ Decreasing Mass

Show predicted peptides also

Start – End	Observed	Mr(expt)	Mr(calc)	ppm	M	Peptide
213 – 223	1444.7472	1443.7400	1443.7510	-7.65	1	R.EIHEFPFEKIR.S
224 – 236	1629.7533	1628.7460	1628.6743	44.0	0	R.SYFQTDFDSYNSR.L
237 – 252	1893.0021	1891.9948	1892.0189	-12.7	2	R.LTQLKDKLDNAIFSMR.K
242 – 252	1309.7211	1308.7138	1308.6496	49.1	1	K.DKLDNAIFSMR.K
242 – 252	1325.7092	1324.7020	1324.6445	43.4	1	K.DKLDNAIFSMR.K + Oxidation (M)
254 – 269	1774.9748	1773.9675	1773.9294	21.5	1	K.AIDTVKENSANLQIMK.Q
275 – 283	1158.6788	1157.6715	1157.6153	48.6	1	R.QLKEQQTQR.L
310 – 318	1110.5677	1109.5605	1109.5063	48.8	0	R.TMESVEWTK.K
310 – 319	1238.6544	1237.6472	1237.6013	37.1	1	R.TMESVEWTKK.E
310 – 322	1620.8302	1619.8230	1619.7977	15.6	2	R.TMESVEWTKKEPR.Q
330 – 360	3784.9471	3783.9398	3783.7703	44.8	2	R.FVDPRNFNNYVNNTDTMMSMFEKVLMMNLLR.S
335 – 352	2199.8879	2198.8806	2198.9071	-12.0	0	R.NFNNYVNNTDTMMSMFEK.V
335 – 352	2215.9072	2214.8999	2214.9020	-0.95	0	R.NFNNYVNNTDTMMSMFEK.V + Oxidation (M)
335 – 352	2231.9125	2230.9052	2230.8969	3.70	0	R.NFNNYVNNTDTMMSMFEK.V + 2 Oxidation (M)
396 – 405	1291.6253	1290.6180	1290.5629	42.7	2	R.RGYRGDEFYE.-

Fragment C

1 MDINKPGWNQ SDQQATAYDP NQQQYYGDGS TYYDPDQAVD PNQAYYPDPN
51 TYPDAAAYYG YGQDGQAYPQ DYAQDPNQAY YADPNAYQDP NAYTDPNAYV
101 DPNAYQDPNA YVDPNNYTDP NAYYGYGQDG QAYPQDYAQD PNQAYYADPN
151 AYQDPNAYTD PYYVTSTDPN AYYGQVDNVP ALEASDLAYE VTPQEQAAEQ
201 ELFSEPETKV IR**EIHEFPFE** **KIRSYFQTDF** **DSYNSR**LTQL K**DKLDNAIFS**
251 **MR**KAIDTVKE NSANLQIMKQ NFERQLKEQQ TQRLTSNTDA EKIGAKINQL
301 EERMQRLSRT MESVEWTKKE PRQEQFDPRF VDPRNFNNYV NNTDTMMSMF
351 EKVLMMNLLR STTPVQPPVQ YFTPQPLTAS PRPVYEEPIS ASFRRRGYRG
401 DEFYE

Unformatted sequence string: **405 residues** (for pasting into other applications).

Sort peptides by ◉ Residue Number ○ Increasing Mass ○ Decreasing Mass

Show predicted peptides also

Start – End	Observed	Mr(expt)	Mr(calc)	ppm	M	Peptide
213 – 221	1175.5493	1174.5421	1174.5659	-20.3	0	R.EIHEFPFEK.I
213 – 223	1444.7276	1443.7203	1443.7510	-21.2	1	R.EIHEFPFEKIR.S
224 – 236	1629.6409	1628.6337	1628.6743	-25.0	0	R.SYFQTDFDSYNSR.L
242 – 252	1309.6368	1308.6296	1308.6496	-15.3	1	K.DKLDNAIFSMR.K
242 – 252	1325.6266	1324.6193	1324.6445	-19.1	1	K.DKLDNAIFSMR.K + Oxidation (M)
244 – 252	1066.5266	1065.5194	1065.5277	-7.83	0	K.LDNAIFSMR.K
244 – 252	1082.4970	1081.4897	1081.5226	-30.4	0	K.LDNAIFSMR.K + Oxidation (M)

Figure S4. PMF results analyzed by MASCOT for protein bands of fragments C and B shown in Fig. S2. Aa sequence (upper) and peptides (lower) identified are colored red. Mr (expt) and Mr (calc) show expected monoisotopic Mr and expected averaged Mr, respectively.

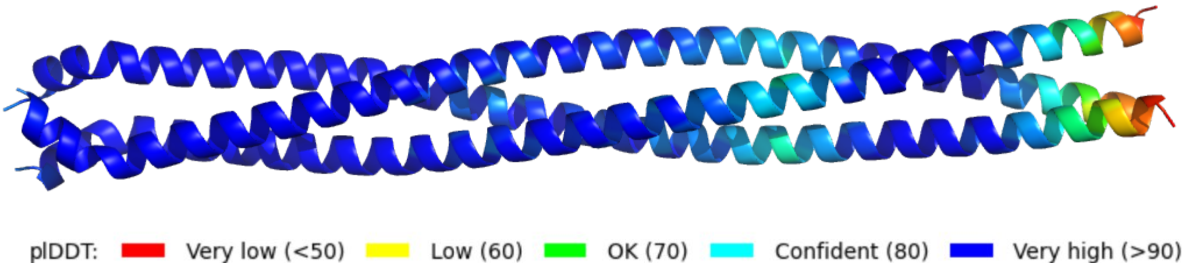

Figure S5. Trimer formation of region II (residues 218–320) predicted by AlphFold2. Prediction was performed using ColabFold v1.5.5 [53]. Residue-specific pLDDT scores are shown by the indicated colors. Residues 218 through 320 are shown from left to right.
